# Defect-induced local variation of crystal phase transition temperature in metal-halide perovskites

**DOI:** 10.1038/s41467-017-00058-w

**Published:** 2017-06-26

**Authors:** Alexander Dobrovolsky, Aboma Merdasa, Eva L. Unger, Arkady Yartsev, Ivan G. Scheblykin

**Affiliations:** 1 Chemical Physics and Nano Lund, Lund University, Box 124, Lund, 22100 Sweden; 2 Helmholtz-Zentrum Berlin GmbH, Institut fur Silizium Photovoltaik, Kekuléstrasse 5, Berlin, 12489 Germany

## Abstract

Solution-processed organometal halide perovskites are hybrid crystalline semiconductors highly interesting for low-cost and efficient optoelectronics. Their properties are dependent on the crystal structure. Literature shows a variety of crystal phase transition temperatures and often a spread of the transition over tens of degrees Kelvin. We explain this inconsistency by demonstrating that the temperature of the tetragonal-to-orthorhombic phase transition in methylammonium lead triiodide depends on the concentration and nature of local defects. Phase transition in individual nanowires was studied by photoluminescence microspectroscopy and super-resolution imaging. We propose that upon cooling from 160 to 140 K, domains of the crystal containing fewer defects stay in the tetragonal phase longer than highly defected domains that readily transform to the high bandgap orthorhombic phase at higher temperatures. The existence of relatively pure tetragonal domains during the phase transition leads to drastic photoluminescence enhancement, which is inhomogeneously distributed across perovskite microcrystals.

## Abstract (web summary)

Understanding crystal phase transition in materials is of fundamental importance. Using luminescence spectroscopy and super-resolution imaging, Dobrovolsky et al. study the transition from the tetragonal to orthorhombic crystal phase in methylammonium lead triiodide nanowires at low temperature.

## Introduction

Recent surge of worldwide attention to organometal halide perovskite (OMHP) materials has already led to solar cells with energy-conversion efficiencies more than 20%^1–3^. These solution-processed materials demonstrate long charge carrier diffusion lengths, low recombination rates, and high absorption coefficients in the whole visible range^3–5^. Because of high luminescence quantum yield, this class of direct bandgap semiconductors is also very attractive for optoelectronic applications such as lasers and light-emitting devices^6–8^.

Although OMHP-based optoelectronics in terms of the achieved efficiencies appears to be a mature technology, the research community is still at the beginning of understanding the complex interplay between excitons, free charge carriers, and various types of structural and electronic defect states^9^, determining the properties of these materials^10–18^. Moreover, electronic properties of OMHPs are sensitive to light and environment. Light can induce not only defect formation, ion migration, and material degradation, but also in some conditions healing of defect states revealed as an improvement of electrical characteristics and enhancement of photoluminescence (PL)^14, 19–22^.

An additional difficulty is that OMHP films are essentially inhomogeneous at the nanoscale and microscale, which greatly affects the observed electronic properties and makes them strongly dependent on the sample preparation method^21–29^. Unmasking the inherent properties from the ensemble averaged response and understanding the effects of nanoscale morphology and crystal structure is an important task for spatially resolved spectroscopy methods and spectroscopy of individual submicrometer crystals^21, 22, 24, 30–33^.

Methylammonium lead triiodide (CH_3_NH_3_PbI_3_ or MAPbI_3_) adopts a perovskite-type crystal structure^18^. It is cubic at temperatures above 330 K, tetragonal between 330 K and ~160 K, and finally transforms to a low-symmetry orthorhombic crystal structure below 160 K^34^. These first-order structural phase transitions lead to deformation of the PbI_6_ octahedra, which alters the electronic and optical properties of the material significantly^35–38^.

Although the tetragonal-to-orthorhombic phase transition of MAPbI_3_ has been described in numerous studies, there is ambiguity in the phase transition temperature. Some studies report that the transition is abrupt, some that it is prolonged over a temperature interval as large as several tens of degrees Kelvin^7, 10–12, 36, 39^. These, and other remarkable features of the phase transition in MAPbI_3_, were recently discussed in relation to possible strain induced by the grain boundaries in polycrystalline films^33^. However, the overall understanding that would coherently unite all observations is still missing.

The PL quantum yield of OMHPs increases upon cooling^7, 10, 11^. Besides this general trend, the PL often becomes especially intense when cooling or heating across the tetragonal-to-orthorhombic phase transition. Although observed in several publications (e.g.,^7, 33^), the origin of this phenomenon remains unclear. Here we use the PL enhancement effect as a reporter on the processes during the crystal phase transition in MAPbI_3_.

In order to shed light on these phenomena, we studied the temperature dependence of PL of highly crystalline individual nanowires (NWs) and microplates of MAPbI_3_. The preparation method used here is known to result in well-defined highly crystalline nanowires, microrods and microplates, as was previously characterized by X-ray diffraction, scanning electron microscopy (SEM), and transmission electron microscopy (TEM)^8^. By studying a number of well-defined individual objects we removed the uncertainty due to ensemble averaging in inhomogeneous films and the effect of grain boundaries in polycrystalline samples. Super-resolution luminescence localization microscopy allowed us to follow the phase transition in individual MAPbI_3_ NWs with spatial resolution below the light diffraction limit.

We show that presence of defects controls the phase transition temperature of each particular region of the crystal. We directly observed the formation of domains of different crystal phases within individual NWs and their coexistence in the phase transition range, visible as a patterned (or spotty as we will call it) PL spatial distribution in what appears to be structurally homogeneous NWs. We assign the drastic PL enhancement in the phase transition temperature interval to freezing out the most defected regions of the crystal to the orthorhombic phase. In other words, we propose that the phase transition temperature in MAPbI_3_ perovskite depends on the concentration of certain defects, which spatially varies in the material at the nanoscale. Our model is based on the theory of phase transitions in solids^40, 41^, which has not been applied to metal halide perovskites yet. The model qualitatively explains the temperature dependence of the PL properties and the spread of the phase transition over the large temperature interval. Thus, our results consolidate apparently opposing literature data concerning the phase transition temperature.

## Results

### Overview of temperature-dependent PL

**Fig. 1:**
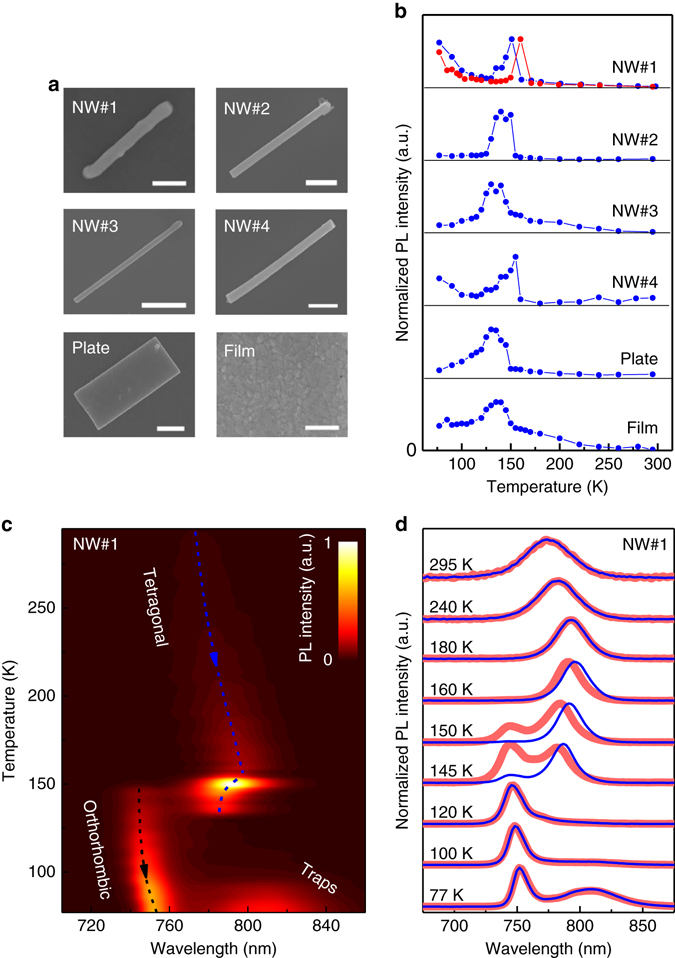
Methylammonium lead triiodide perovskite samples and their temperature-dependent PL. SEM and temperature-dependent PL of individual methylammonium lead triiodide perovskite NWs (NW#1, NW#2, NW#3, NW#4), microplate, and a film. **a** SEM images of the different samples; the *scale bar* is 1 μm. **b** Variation of the normalized spectrally integrated PL intensity of the samples under cooling and heating (*blue circles* and *red circles*, respectively). **c** *2D map* (data were interpolated) of PL spectrum vs. temperature under cooling for NW#1. The *dashed lines* show the evolution of the emission peaks with temperature. **d** Normalized PL spectra of NW#1 at different temperatures during cooling (*blue lines*) and heating (*red lines*). PL spectra are vertically shifted for clarity

Figure 1 shows SEM images of four NWs, one microplate, and a polycrystalline film (a) together with the temperature dependences of their PL intensity (b). All NWs and the plate possess well-defined rectangular shapes determined by their crystallographic axes, which is in great contrast to the thin film consisting of randomly shaped grains of 100 – 300 nm in size. All samples possessed the PL enhancement effect in a certain temperature region around 150 K. The relative amplitude of the PL enhancement was larger and the temperature region of the enhancement was narrower for individual NWs when compared to the individual plate and the film sample.

Figure 1c and d show temperature-dependent PL spectra of NW#1 while similar data for the other objects are given in Supplementary Figs. 9–13. At temperatures from 295 to 160 K, PL of NW#1 possessed one emission peak corresponding to the tetragonal phase. This peak was observed to monotonically shift toward longer wavelengths upon cooling, which is in agreement with the anomalous Varshi trend with a positive thermal expansion coefficient^42^. Upon cooling from 160 to 140 K (around the phase transition temperature), the PL peak of the tetragonal phase started to shift toward shorter wavelengths while exhibiting a dramatic flash-like increase of the PL intensity, reaching its maximum at ~150 K. Around this temperature, the PL peak attributed to the orthorhombic phase appeared and its intensity increased upon further cooling^11, 12^. Thus, in the temperature region from 150 to 130 K, the PL bands of both the tetragonal and orthorhombic phase were observed showing the coexistence of both phases within the individual NWs of high crystallinity over at least a 20 K temperature interval.

The PL intensity from the tetragonal phase decreased until it vanished around 130 K, indicating the absence of the tetragonal phase, which is in agreement with previous X-ray diffraction studies^12^. At temperatures below 100 K, a low-energy feature appeared (emission peak around 820 nm, see also Supplementary Fig. 3), that has been previously attributed to emission from trap states^7, 10, 12^.

Figure 1d shows the normalized spectra for both heating and cooling cycles. One can see an apparent thermal hysteresis of the PL peak position of the tetragonal phase and its emission intensity^31^, which is similar to the recently reported hysteresis of the carrier mobility in perovskite microplates near the phase transition temperature^43^.

### Spatially resolved PL spectra at room temperature

**Fig. 2:**
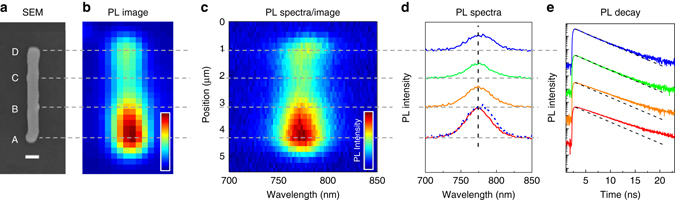
Spatially resolved PL spectra and PL decay kinetics of the nanowire NW#1 at 298 K. **a** SEM image; the *scale bar* is 500 nm. **b** *Luminescence image*. **c** PL emission intensity as a function of wavelength and the emission position along the nanowire axis. **d** The PL spectra collected from the regions marked by the *horizontal lines* A–D, spectrum of region D was scaled and overlaid with the spectrum of region A (*dashed line*) to show the spectral shift and broadening. **e** Time-resolved PL decay profiles collected from the same regions of NW#1; the *dashed lines* are the guides showing an exponential decay with the lifetime of 2.5 ns. *Horizontal dashed lines* are eye guides to mark the regions of the nanowire where the particular properties were measured

Now let us have a look at how the PL properties of NW#1 change along its length. Spatially resolved PL spectra and PL decay kinetics at room temperature are presented in Fig. 2. The SEM image shows that NW#1 had a uniform rod-like shape with almost constant thickness and without visible grain boundaries. Therefore, NW#1 should absorb the excitation light uniformly along its length. However, the PL intensity and PL lifetime were observed to slightly differ from one spatial location to the other. Higher PL intensity corresponded to a longer PL lifetime. Since the excitation conditions were the same, a longer PL lifetime is indicative of a smaller concentration of non-radiative recombination centers in the bright region A of NW#1 in comparison to the dimmer regions B, C, and D in Fig. 2. PL spectra of the darker regions were slightly broader than those of the bright region A. The spectrum from region D also appeared to be red-shifted by 8 nm in comparison to the one from the high PL intensity region A.

### Spatial and spectral inhomogeneity of PL during transition

Luminescence microscopy allowed us to see evolution of the PL intensity distribution over the NW during the phase transition. The Supplementary Information contains a video composed of PL images during the cooling–heating cycle between 295 and 77 K, where a clear spatial redistribution of PL is observed in the temperature region between 160 and 130 K.

**Fig. 3:**
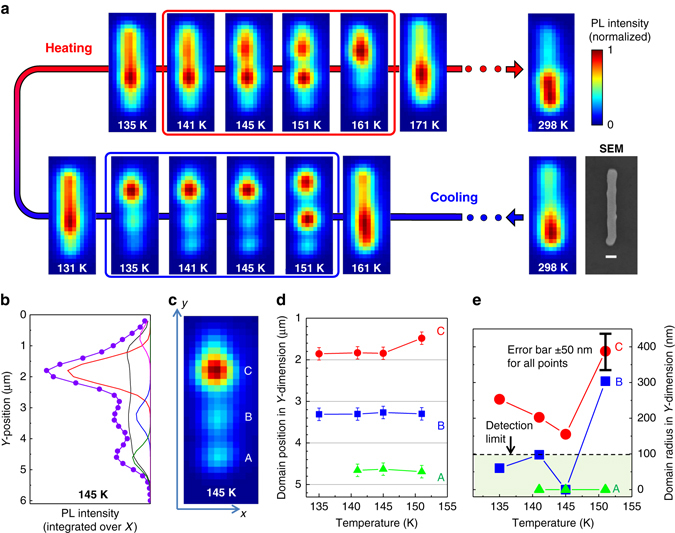
Evolution of the PL intensity distribution over the nanowire NW#1. **a** PL images of the NW at different temperatures during the cooling and heating cycles; the *scale bar* shown in SEM image is 500 nm. **b** Fitting of the PL intensity distribution at 145 K along the NW (*y-axis*) with four Gaussian peaks representing four emitting regions of the tetragonal phase and a background (*black line*) coming from the orthorhombic phase (see Supplementary Note 3, Supplementary Figs. 5, 6). The *violet dots* represent the original data and the *violet line* the overall fit. **c** Luminescence image of the NW at 145 K showing the tetragonal domains A–C; the *color chart* is the same as for images in **a**. **d**, **e** Temperature variation of the position and the characteristic radius of the domains A–C along the *y-axis* as a function of temperature in the cooling cycle (Supplementary Note 4). All data points in **d** have the same absolute error bar ( ± 50 nm) as, for the sake of clarity, is shown for one data point only. The *error bars* in **d**, **e** were calculated taking into account the noise of the data and uncertainty of the fitting/deconvolution procedure

Figure 3 and the Supplementary Movie nicely show that the PL of the investigated NWs becomes spotty across the phase transition temperature region. Upon further cooling, the relative intensities of the emission spots were drastically changing (see Fig. 3 and the video in Supplementary Information). Above or below the phase coexistence region (T > 160 K or T < 130 K), the PL appeared much more uniform over the length of the NW. More examples of spotty PL patterns appearing in the phase transition region can be found in Supplementary Figs. 7, 9–12.

**Fig. 4:**
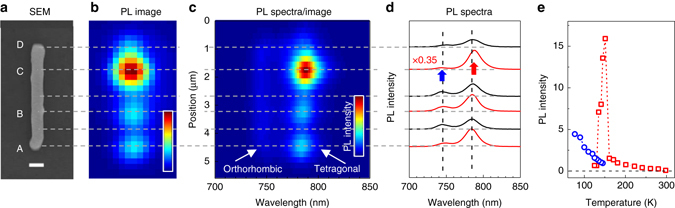
Spatially resolved PL spectra of the nanowire NW#1 at 145 K. **a** SEM; the *scale bar* is 500 nm, and **b** *luminescence images*. **c** The PL emission intensity as a function of wavelength and emission position along the NW *y-axis*. The luminescence belonging to the tetragonal phase (*λ* = 780 nm) is *spotty*, while the orthorhombic phase emission (*λ* = 740 nm) is *uniform*. **d** The corresponding PL spectra collected from the different regions of the NW. **e** Temperature dependences of the intensities of PL bands associated with the tetragonal (*red squares*) and orthorhombic (*blue circles*) domains at spatial region C of NW#1 (spectral positions of the PL bands are marked on **d** by *red arrows* and *blue arrows*, respectively). *Horizontal dashed lines* are eye guides to mark the regions of the NW where the particular properties were measured

Figure 4 shows the variation of the PL intensity and spectra along the axis of NW#1 at 145 K—the temperature when the spotty emission is very clear. The PL spectra contain two emission bands associated with the different crystal phases obviously coexisting in the very same NW in the 130 – 150 K temperature range. We found that the intensity of the lower energy emission peak (tetragonal phase) was much higher for the bright spots in comparison to that in the dark regions of the same wire. It is even better illustrated by the spectral image in Fig. 4c, where the PL from the tetragonal phase was clearly stemming from three distinct locations, while the emission of the orthorhombic phase was evenly distributed along the NW length. The spectral images taken during the cooling/heating cycle can be found in Supplementary Fig. 4.

This shows that the PL enhancement is due to the increasing PL intensity from the tetragonal phase only. The spatially resolved measurements show that the PL of the tetragonal phase stems from several small regions within the NW, which positions and sizes will be characterized further below using super-resolution localization microscopy approach.

We employed the concept of Super-resolution Luminescence Micro Spectroscopy (SuperLuMS) to combine super-resolution imaging with spectral measurements^31, 44^. The idea here is to fit the emission pattern with a combination of several Gaussian peaks, representing the emission from the localized tetragonal domains, and a background emission more uniformly distributed over the NW (Supplementary Note 3). The latter, as we have discussed above, was coming from the orthorhombic phase, and we were able to determine its contribution using the spectral information from the same experiment (Supplementary Figs. 5, 6).

Figure 3 shows the appearance of NW#1 PL images at different temperatures and the results of the fitting procedure applied to the image at 145 K integrated over the NW width. As one can see, the emission pattern can indeed be modeled by an almost uniform background estimated from the orthorhombic phase emission plus four Gaussian peaks characterized by their width and the position of the maximum along the NW (*y*-axis). The position of the peak gives the PL intensity-weighted center of the tetragonal domains, while the width of the peak can be deconvoluted in order to characterize the domain radius (Supplementary Note 4). The temperature dependencies of the domain center positions and sizes demonstrate that the domains were decreasing in size upon cooling. Domains B and C were initially ~300–400 nm in radius and decreased below the reliable detection limit (100 nm) upon further cooling. Domain A was consistently smaller than 100 nm.

## Discussion

The PL quantum yield is determined by the ratio of the efficiencies of the radiative and non-radiative recombination processes. Although the radiative rate in semiconductors is dependent on the concentration of charge carriers^9^ (since the excitation conditions were kept constant in our experiments), we conclude that the PL enhancement at the phase transition temperatures must mainly be due to a drastic reduction of the non-radiative rates in domains of the tetragonal phase. Non-radiative processes have been discussed in numerous literature reports and attributed to PL quenching on trap states^18^. In order to explain the PL enhancement, we then need to assume that either the concentration of traps or the efficiency of charge carrier transport toward the traps dramatically decrease in the phase transition temperature region. Below we will argue that both effects most probably take place.

Indeed, it is known that when a solid transforms from one crystal phase to another, the nucleation process depends on the local properties of the material and in particular on the presence of structural and chemical defects^45–48^. Theoretically, for first-order phase transitions occurring between phases of different symmetry it is the so-called random local field defects that are especially important^40, 41^. Depending on the symmetry of such a defect, it can stimulate the transition toward the structure of either higher or lower degree of symmetry. In other words, depending on the symmetry of the defects, the phase transition temperature can increase or decrease. Difference in the local concentration of such defects can result in the phase transition occurring at different temperatures at different spatial locations in the sample. On the basis of this general phenomenon, we present a model below which can qualitatively explain our experimental observations and consolidate the literature results.

**Fig. 5:**
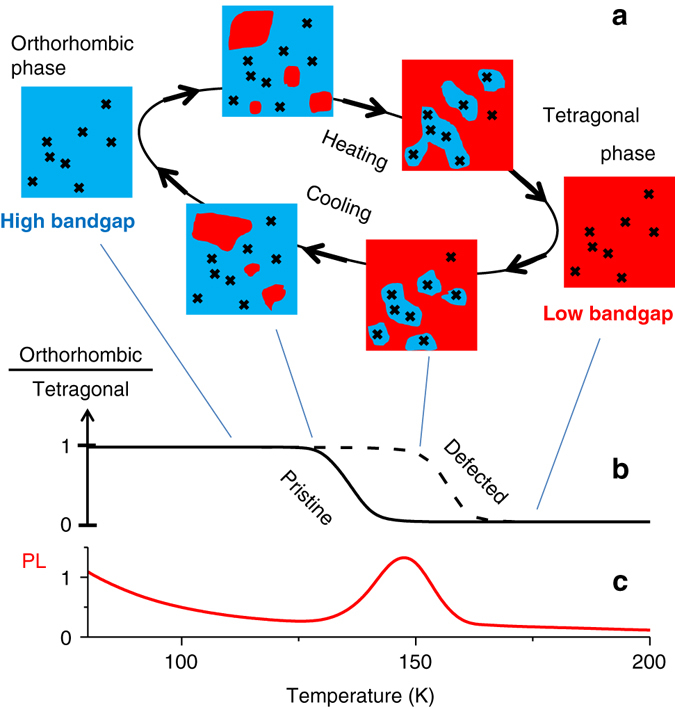
The proposed model of the phase transition in a perovskite crystal with defects. **a**, **b** The *cartoon* shows temperature dependence of the ratio **b** between orthorhombic (*blue regions* in **a**) and tetragonal (*red regions* in **a**) phases in a nanocrystal containing defects (*black crosses* in **a**). We assume that the transition temperature depends on the local concentration of defects and that the defects that quench PL also stabilize the low symmetry orthorhombic phase at higher temperature. That is why the domains containing such defects (*black crosses*) transfer to the orthorhombic phase at higher temperature (*dashed line* in **b**). The bandgap of the orthorhombic phase is larger than that of the tetragonal phase, leading to trapping of charge carriers in the pristine tetragonal phase possessing a smaller defect concentration. All these explain the PL intensity enhancement at the phase transition temperature region illustrated in **c**

Figure 5 shows a schematic picture of our model. Let us consider the cooling cycle. We assume that the temperature of the transition from tetragonal to orthorhombic phase is higher for domains with high defect concentration (~160 K) and lower for domains with a lower defect concentration (~140 K). Therefore, the defects causing PL quenching stimulate formation of the low-symmetry orthorhombic phase at higher temperatures, or, in other words, they make the low-symmetry phase stable at higher temperature. Thus, at temperatures slightly below 160 K most of the crystal domains with large concentration of such defects must already be in the high-bandgap orthorhombic phase, while most of the relatively clean material remains in the more symmetric tetragonal phase of the lower bandgap. When illuminated at 485 nm, both phases are excited and most of the mobile charges should migrate to low-bandgap tetragonal domains due to the funneling effect, leading to a higher PL quantum yield because these domains possess lower defect concentration^12^. We also experimentally observed funneling of charge carries from the orthorhombic to the tetragonal domains^12^ as an increase in PL intensity in the tetragonal phase with the time constant 0.4 ns (Supplementary Fig. 8). Thus, the charge carrier funneling and their confinement in the tetragonal phase domains, which possess a lower concentration of PL quenching defects, explains the emission enhancement effect.

As soon the entire volume of the crystal is transformed to the orthorhombic phase, both effects are gone and the PL intensity drops down. If we heat the sample up, the exact same sequence of events will lead to the similar PL intensity/spectrum behavior but in the reverse order (Fig. 5).

Since breaking into nanodomains of different phases is a random process, although dependent on the concentration and nature of local defects, it is probably not so surprising to observe NW#1 exhibiting the strongest PL enhancement in spatial segment C (Fig. 4), which in fact possessed the lowest PL intensity at room temperature compared to the other regions of the same NW (Fig. 2). It shows that eliminating contributions of the defected regions to PL of the crystal can lead to drastically different spatial emission patterns reflecting local inhomogeneities of the defect concentration and peculiarities of charge diffusion at the nanoscale.

This simple model where a very efficient carrier diffusion over the whole NW is assumed has some difficulties to explain why the PL was gradually decreasing upon further cooling instead of being quasi-constant in the temperature region where pure tetragonal domains are present in the dominant orthorhombic phase (from 150 to 135 K for the cooling cycle). Indeed, due to the charge carrier funneling, even a very small tetragonal domain at, e.g., 135 K should be as bright as the same domain but of a larger size at 150 K. This is, however, not the case as illustrated for peak C in Fig. 4e. To explain this, we propose that the charge carrier funneling distance (or migration volume of charge carries) is substantially smaller than the size of the whole NW. In this case, recombination in a gradually increasing volume of the orthorhombic phase will compete with funneling to the gradually decreasing tetragonal inclusions leading to a decrease in the emission intensity of the latter. In addition, decreasing the domain size may give rise to strain-induced, non-radiative recombination channels.

We also believe that decreasing of the domain size is the reason of the blue shift of the tetragonal phase PL spectrum when cooling over the phase transition region (Fig. 1c). Note that this shift is the opposite of the shift we observed upon cooling of the sample in the pure tetragonal phase (160 K < *T* < 300 K). Shifting the bandgap toward higher energy means geometrical deformation of the crystal structure toward a smaller Pb–I–Pb bond angle as has been shown theoretically^38, 49^. Variations of the bandgap energies have also been observed experimentally in perovskite samples with different characteristic sizes, where it was related to the surface-induced strain in small crystallites^25, 50^. Thus, in the framework of our model, the closer the temperature is to 140 K (in the cooling cycle), the smaller the sizes of the tetragonal inclusions are. Smaller domain sizes mean larger stress from the bulk orthorhombic phase surrounding the tetragonal nanoinclusions, which results in a blue-shifted PL spectrum. The variety of the domain sizes can also explain the variation of the peak position for both PL bands at the different regions of the NW (see Fig. 4d).

Coexistence of nanodomains of different phases inside intact crystals is the origin of several different phenomena and their temperature hysteresis. For example, it should hinder charge diffusion because of charge trapping in domains of the tetragonal phase, as well as scattering on domain boundaries. This agrees with the decrease in charge carrier mobility in MAPbI_3_ reported around the phase transition temperature of 150 K and its thermal hysteresis^43^. Solar cell devices exhibit a discontinuity in photovoltaic performance, especially in the trend of the open circuit voltage through the phase transition^51^. A very strong thermal hysteresis of optical properties was reported for polycrystalline films of MAPbI_3_ studied by confocal PL microscopy^33^. Even for large single crystals, thermal hysteresis of absorption spectra, mechanical properties, and geometry of large single crystals of organic–inorganic (C_12_H_25_NH_3_)_2_PbI_4_ perovskites was observed^52^, which is in agreement with our results on individual highly crystalline NWs. As it is the usual case for the first-order phase transitions in solids, the nucleation process depends on the mechanical strain in the crystal, which in turn depends on the nucleation. Therefore, the whole process depends on its history leading to hysteresis. In terms of our model, both defected and clean domains of the crystal possess thermal hysteresis, which leads to the hysteresis of the PL enhancement as well (Fig. 1b).

Besides the current study on the single object level, observation of PL enhancement in the phase transition region has been reported for thin film samples in several publications^7, 33^. The extent of the enhancement and the temperature interval of the enhanced PL vary a lot from sample to sample and even from one individual crystal to another (Fig. 1). However, the general trend that seems to be present is that higher disorder and polycrystallinity result in a less sharp PL enhancement and a broader temperature region of enhanced PL where both phases coexist.

There is a great variety of intrinsic defects (vacancies, substitutions, and interstitials) possible in MAPbI_3_^26^. In principle, defects influencing the phase transition can be of any type and are located in the bulk or on the surface of the crystal. The phase transition may even be induced/prevented by a grain boundary in a polycrystal. The only criterion we can use to select possible defects crucial for the phase transition in the samples possessing PL enhancement is that they, in comparison to other defects, must be the most important channels for non-radiative recombination. If we assume quenching by Shockley–Read–Hall non-radiative recombination, the defects should have relatively deep levels in the bandgap. According to ref. ^26^ such deep traps can be I_MA_, I_Pb_ and Pb_I_ substitutions and Pb_i_ interstitials.

Note that a small extent or even the absence of PL enhancement, which some reports seem to show^53^, by no means undermines the generality of our model. It only implies that the defects that were determining the phase transition dynamics in those samples were not the ones that were crucial for their PL quantum yield. In this case the local PL yield becomes insensitive to local crystal phase.

In summary, we present a spatially resolved luminescence spectroscopy study of PL temperature dependence for individual MAPbI_3_ NWs. We confirm the coexistence of domains of both tetragonal and orthorhombic phases in single NWs of high crystallinity across the phase transition temperature region between 160 and 140 K where a strong enhancement by one order of magnitude of the PL quantum yield at ~150 K was observed. Hysteresis of the PL spectrum and PL quantum yield was also seen. The largest observed crystal phase domains possessed sizes in the range of 100–500 nm as estimated by super-resolution analysis of the luminescence images.

We propose that the phase transition temperature in OMHPs is dependent on the defect concentration and their type. Using this concept we can readily explain the PL enhancement effect by suggesting that crystal regions with higher defect concentration transform to the orthorhombic phase at higher temperature upon cooling compared to more pristine crystal regions. Thus, transforming the defected regions to the orthorhombic phase increases PL intensity dramatically. This is because PL stems mostly from low-defect density tetragonal inclusions harvesting charge carriers from higher-energy orthorhombic phase domains. During cooling, physical sizes of the tetragonal inclusions become smaller, which increases the internal strain leading to the observed blue shift of the PL spectrum.

Dependence of the phase transition temperature on the local defect concentration means dependence on the sample preparation, which explains the lack of consensus in the literature on the value of the transition temperature. We stress that our experiment at the single NW level shows that the spread of the transition temperature over tens of degrees Kelvin is an intrinsic effect for MAPbI_3_ related to defects in general, but not just the consequence of sample polycrystallinity and effects of the grain boundaries. The effect of freezing out defects of a certain type in a particular crystalline phase may lead to ideas of material manipulation based on, e.g., repeatedly running a sample through cooling–heating cycles across the phase transition, which may lead to enhanced optoelectronic properties.

## Methods

### Sample preparation

NWs of methylammonium lead iodide, CH_3_NH_3_PbI_3_, were synthesized using a procedure described in literature (Supplementary Note 1 and Supplementary Figs. 1, 2)^8^. Lead acetate was drop-cast from a 100 mg ml^−1^ solution of PbAc_2_3H_2_O in water onto cleaned glass slides placed on a hot plate at 65 °C. The films were dried for ~30 min and then immersed into a solution of 40 mg ml^−1^ methylammonium iodide in isopropanol. The sample was left to convert for at least 24 h to form a sample of NWs and nanoplatelets shown in Supplementary Fig. 1. Individual NWs were mechanically transferred onto Si/SiO_2_ substrate for PL microspectroscopy measurements. To prepare the thin film sample, we dissolved methylammonium iodide and lead iodide precursors in dimethylformamide (1:1 molar ratio). 100 μl of this solution was then spin-cast onto a glass coverslip for 1 min at 1500 rpm followed by annealing on a hot plate for 45 min at 80 °C.

### Temperature-dependent PL microspectroscopy measurements

Temperature-dependent PL microspectroscopy measurements were performed in a variable temperature liquid nitrogen cryostat using a home-built, wide-field luminescence microscope based on Olympus IX71 (Supplementary Note 2). Diode laser (485 nm) in CW mode and pulse mode was used as the excitation source. The luminescence image of single NWs was obtained by ×40 objective lens and detected by the charged-couple device camera after passing through a transmission grating in order to obtain their spectra. Time-resolved PL was measured using a time-correlated single-photon counting system together with excitation by a 485 nm diode laser operating in the pulsed mode. The luminescence was detected by a fast avalanche photodiode coupled with a PicoQuant PicoHarp 300 counting module.

### Data availability

The data that support this study are available from the corresponding author upon request.

## Electronic supplementary material

Supplementary Information

Supplementary Movie 1

Peer Review
