# Supplementary material for: Defect-induced local variation of crystal phase transition temperature in metal-halide perovskites
Source: Nat Commun. 2017 Jun 26;8:34. doi: 10.1038/s41467-017-00058-w (PMC5484711; doi:10.1038/s41467-017-00058-w)
Supplement: Supplementary file 1 — Supplementary Information [file 41467_2017_58_MOESM1_ESM.pdf]

File name: Supplementary Information
Description: Supplementary Figures, Supplementary Notes and Supplementary References

File Name: Supplementary Movie 1
Description: The movie is composed of photoluminescence images of the nanowire NW#1 during the cooling – heating cycle between 295 and 77 K. A clear spatial redistribution of photoluminescence intensity (spotty luminescence) is observed in the temperature region between 160 and 130 K. The sample temperature is indicated on each frame of the movie.

File Name: Peer Review File
Description:

Supplementary Note 1: Sample preparation and characterization.

Nanowires of methylammonium lead triiodide ($CH_3NH_3PbI_3$ or $MAPbI_3$) were synthesized using a procedure described in literature.[1] Lead acetate was drop-cast from a 100 mg mL^{-1} solution of $PbAc_2$ * 3 H_2O in water onto cleaned glass slides placed on a hotplate at 65°C. The films were dried for about 30 minutes and then immersed into a solution of 40 mg ml^{-1} methylammonium iodide (CH_3NH_3I) in isopropanol (IPA). The sample was left to convert for at least 24 hours to form a sample of nanowires and nanoplatelets shown in Supplementary Figure 1.

The corresponding X-ray diffraction (XRD) pattern is shown in Supplementary Figure 2. The narrow diffraction peaks indicate a high degree of crystallinity and can be attributed to the tetragonal room-temperature phase of $CH_3NH_3PbI_3$. The diffraction peaks were assigned to the different diffraction planes according to the assignment made by Baikie et al.[2] Due to the high crystallinity of the samples, a clear splitting between the peaks is observed that allows the distinction of the often overlapping hk0 and 00l peaks of the tetragonal phase.

It was found that the nanowires had lengths up to several micrometers and diameter varying from 100 to 500 nm with predominantly flat rectangular end facets indicating high-quality single crystal structure (Supplementary Figure 1).

From structural refinement, the lattice parameters of the tetragonal phase were determined to be a = 8.87 Å and c = 12.66 Å in close agreement with the values determined for highly crystalline $PbCl_2$-derived $CH_3NH_3PbCl_xI_{3-x}$ samples.[3] Compared to the highly oriented growth discussed in Ref.3 we observed reflections from all crystal planes due to the random orientation during growth of the nanowires/nanoplates shown in Supplementary Figure 1.

For scanning transmission electron microscopy (STEM) characterization, nanowires were mechanically transferred onto a TEM grid. Individual nanowires were mechanically transferred onto Si/SiO_2 substrate for SEM and PL micro-spectroscopy measurements. For that we used a piece of a soft paper to take the crystals from the original substrate and release some of them to the clean substrate for PL micro-spectroscopy.

To prepare the thin film sample we dissolved methylammonium iodide and lead iodide precursors in dimethylformamide (1:1 molar ratio). 100 μL of this solution was then spin-cast onto a glass coverslip for 1 minute at 1500 rpm followed by annealing on a hotplate for 45 minutes at 80°C.

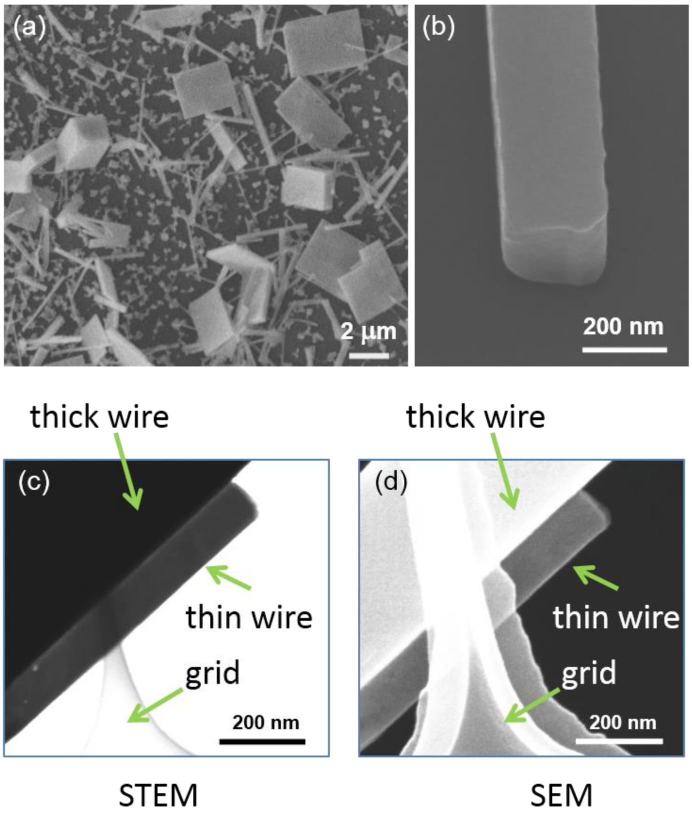

Supplementary Figure 1: Electron microscopy images of nanowires and microplates. (a) SEM image of as-prepared $CH_3NH_3PbI_3$ nanowire/microplate sample. (b) 30°-tilted SEM image of single nanowire on Si/SiO_2 substrate. (c,d) STEM and SEM images of the single nanowire, the TEM grid supporting the sample is also visible there.

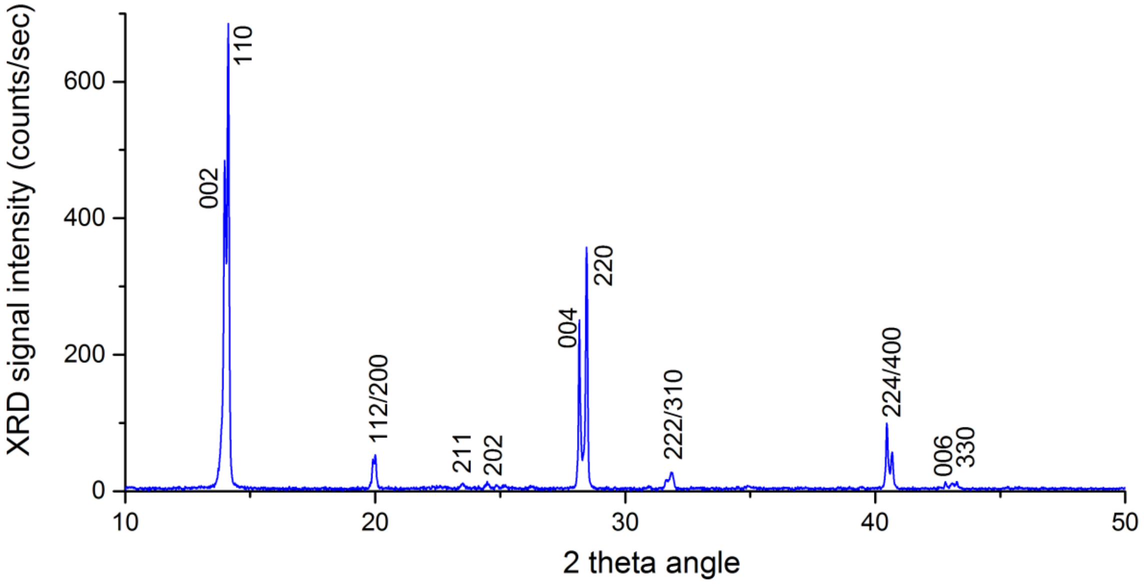

Supplementary Figure 2: X-ray diffractogram with the peak assignment. Data for the nanowire/microplate $CH_3NH_3PbI_3$ sample shown in the SEM image in Supplementary Figure 1.

Supplementary Note 2: Temperature-depended micro-PL setup.

Temperature-dependent micro-PL measurements were performed in a variable temperature liquid nitrogen cryostat using a home-built wide-field fluorescence microscope based on Olympus IX71.[4,5] A 485 nm diode laser (LDH-D-C-485, PicoQuant GmbH) in either CW and pulsed operation modes was used as the excitation source. The fluorescence image of single nanowires was obtained by 40× objective lens (Olympus LUCPlanFL, NA = 0.6) and detected by the CCD camera (ProEM 512B, Princeton Instruments) after passing through a transmission grating in order to obtain their spectra. Time-resolved PL was measured using a time-correlated single-photon counting (TCSPC) system with the 485 nm diode laser in the pulsed mode as the excitation source. The luminescence was detected by a fast avalanche photodiode (APD, Micro Photon Devices) coupled with the PicoHarp 300 (PicoQuant GmbH) counting module.

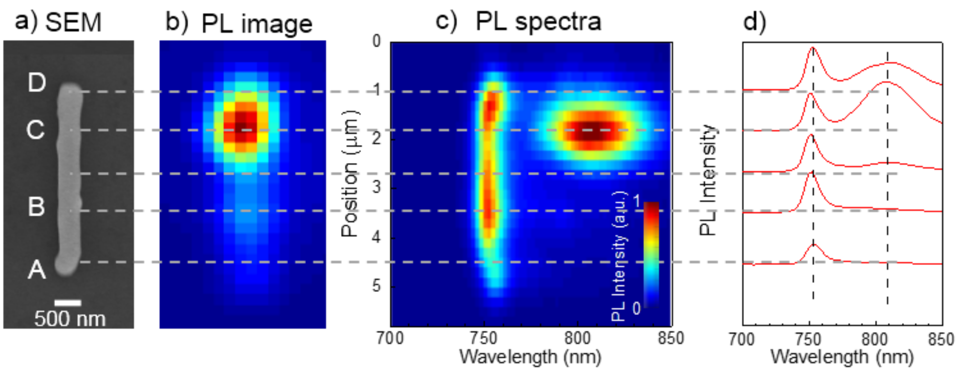

Supplementary Figure 3: Spatially resolved PL spectra of NW#1 at 77K. (a) SEM and (b) fluorescence images. (c) The PL emission intensity as a function of wavelength and the emission position along the nanowire y-axis. (d) The PL spectra collected from the regions marked by the horizontal lines (A-D). The spectral band around 800 nm is assigned to radiative transitions at trapping sites. This emission is very inhomogeneously distributed over the nanowire.

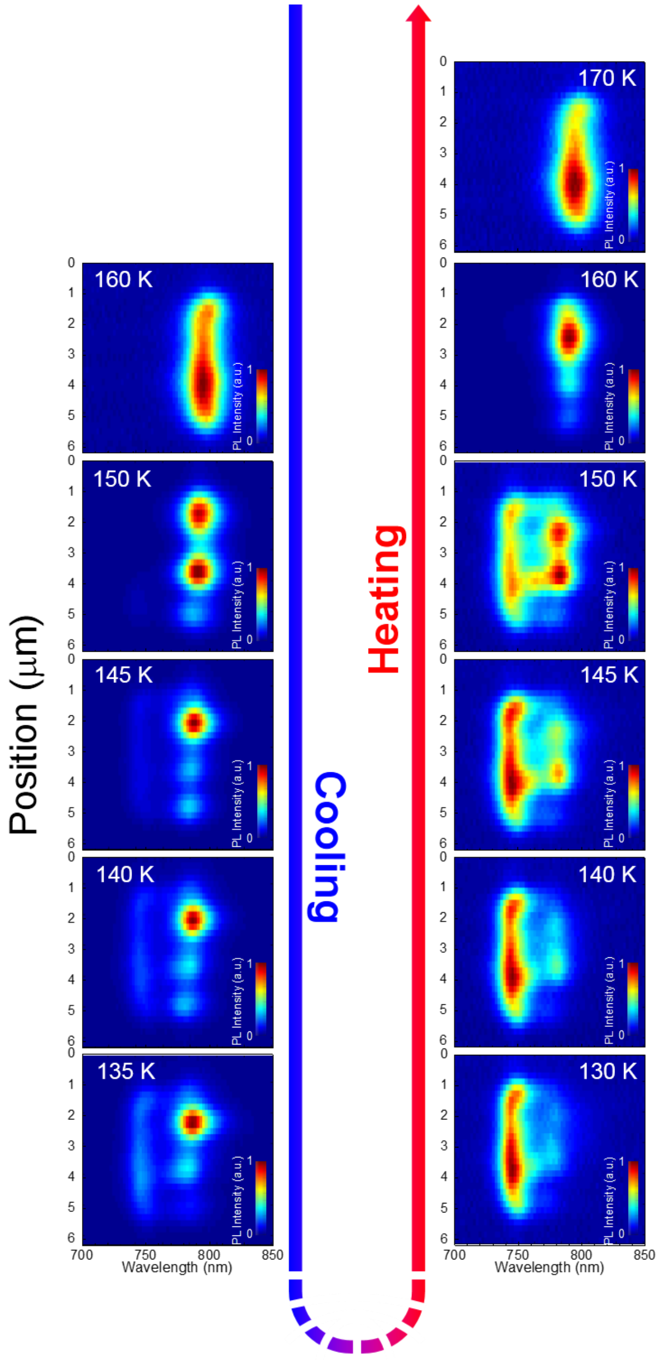

Supplementary Figure 4: Evolution of the PL spectrum over the nanowire length during the phase transition. Evolution of the PL spectrum (horizontal axis) over the length of NW#1 (vertical axis) during the phase transition. The spectral band at 740 nm belongs to the orthorhombic phase and the band at 770 nm belongs to the tetragonal phase.

Supplementary Note 3: Super-resolution analysis of the "spotty" PL of the nanowire.

Prior to any super-resolution analysis[5,6] we corrected the data for drift of the sample stage between each acquisition for every temperature. We accomplished this by applying a sub-pixel image correlating algorithm. This algorithm simply finds the strongest correlation of all images with respect to the first image where sub-pixel shifts in the x-y plane are allowed. Each pixel has an effective size of 200 nm and sub-pixel shifts were made with increments of 25 nm. In order to apply such a correction each raw image was converted to an artificial image (with the same intensity distribution but across more pixels) where the shifts were made. After the correction, the images were converted back to the original pixel resolution. This procedure doesn't change the number total intensity counts but just redistributes them in the original image. We estimated the accuracy of this procedure of about 150 nm. It was not better than that because the images contained just a very few luminescent objects and the spatial intensity distribution was changing within them.

The luminescence image of the NW#1 was integrated over the axis perpendicular to the nanowire length (x-axis) because we were interested in the distribution of the emission regions along the wire. After that we applied a sum of three to four 1D Gaussian functions to fit the PL intensity distribution. However, such a procedure was not always successful because a part of the emission in some cases was rather uniformly distributed along the nanowire making fitting only by a Gaussian not appropriate. We were able to obtain information about the background emission from the spectral images as described below.

Once all images were corrected for drift, we first looked at the spectral images taken at the temperatures across the phase transition (T=151 K, 145 K, 140 K, and 135 K). In these images we could first identify if the emission from the orthorhombic phase was present and if so, its intensity distribution along the nanowire (see rectangle marked with red dashed lines in Supplementary Figure 5).

We then extracted the shape of the orthorhombic emission and used it as a background for fitting the peaks observed in the PL image at the corresponding temperature. Constant emission from the orthorhombic phase was not significantly present at 151 K whereas for the other three temperatures it was. For each temperature the shape was scaled and shifted until the best fit was acquired.

When fitting the peaks we applied a 1 D Gaussian fit for each peak according to Eq. 1

$$G(x) = A\, exp^{-\frac{(x-\mu)^2}{2\sigma^2}} \qquad \text{(Eq.1)}$$

where A is the amplitude, μ is the position of the peak and σ a characterization of the peak width (FWHM = 2.35 σ). In all cases, three peaks were visible, however, we applied a fit containing four peaks to see if the residuals improved, and this was the case for T = 145 K and 141 K. However, observing their shapes and position, they could as well have been part of the larger peak or part of the orthorhombic emission. For this reason we only considered the three major peaks seen for all temperatures in Supplementary Figure 6. Here we show the fits for each peak including the scaled orthorhombic emission for each of the temperatures. At T = 151 K no orthorhombic emission is used as a background since this was not present in the PL spectral image (see Supplementary Figure 4). At all four temperatures, three peaks (A, B and C in Figure 3) were visible, however, at T = 135 K we see that the right peak is almost indistinguishable from the orthorhombic emission (black line). Therefore, we did not consider this data point in the results presented in the paper.

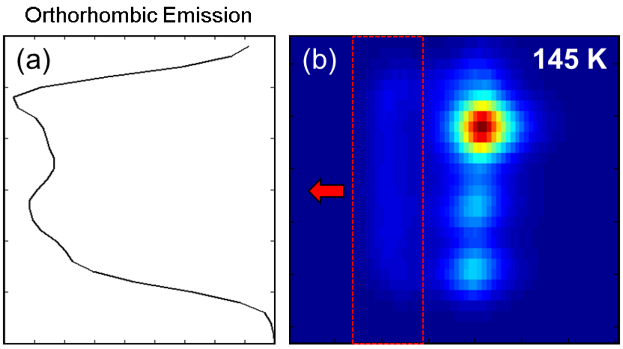

Supplementary Figure 5: Separation of the emission of different crystal phases in a spectral image. (a) shows the shape of the orthorhombic emission integrated between the dotted red lines in (b) which represents the PL spectral image of the nanorod emission at T = 145 K.

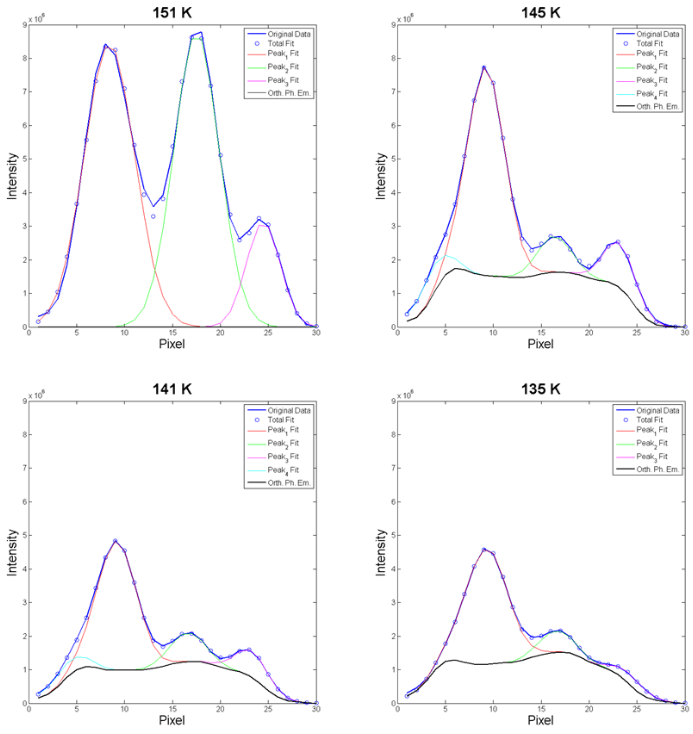

Supplementary Figure 6: **Super-resolution analysis of tetragonal domain emission.** Fit results for the peaks at temperatures 151 K, 145 K, 141 K and 135 K. The blue lines represent the original data and the blue dots the overall fit. The black lines represent the orthorhombic emission shape for each temperature extracted using the PL spectral images. Each peak fit is plotted with a different colour. In this representation the solid black line (orthorhombic phase emission) is the background level to which the Gaussian peaks were added.

Supplementary Note 4: Estimation of size of objects smaller than the diffraction limit by a deconvolution procedure.

The image detected by the CCD camera is a convolution of the real object and the point spread function (PSF) of the microscope. For our microscope, $\sigma_{PSF} \approx 360$ nm at a peak emission wavelength of 760 nm. Convolution of two Gaussian functions (G1 and G2 with standard deviations σ_1 and σ_2) is a special case which can be calculated analytically:

$$G1 \otimes G2 = G3$$

where G3 is also a Gaussian function with the standard deviation $\sigma_3^2 = \sigma_1^2 + \sigma_2^2$

If G3 represents the experimental image of an object and G1 is the PSF, the following equation gives the standard deviation of a Gaussian which represents the real size of the object:

$\sigma_{object} = (\sigma_{image}^2 - \sigma_{PSF}^2)^{1/2}$

In the paper we use σ_{object} as the characteristic radius of the object. Of course, this procedure only provides a rough estimation of the object size because we do not know *a priory* the real shape of the object. Also, the object has more than likely an asymmetric shape where we only apply the fit in one dimension.

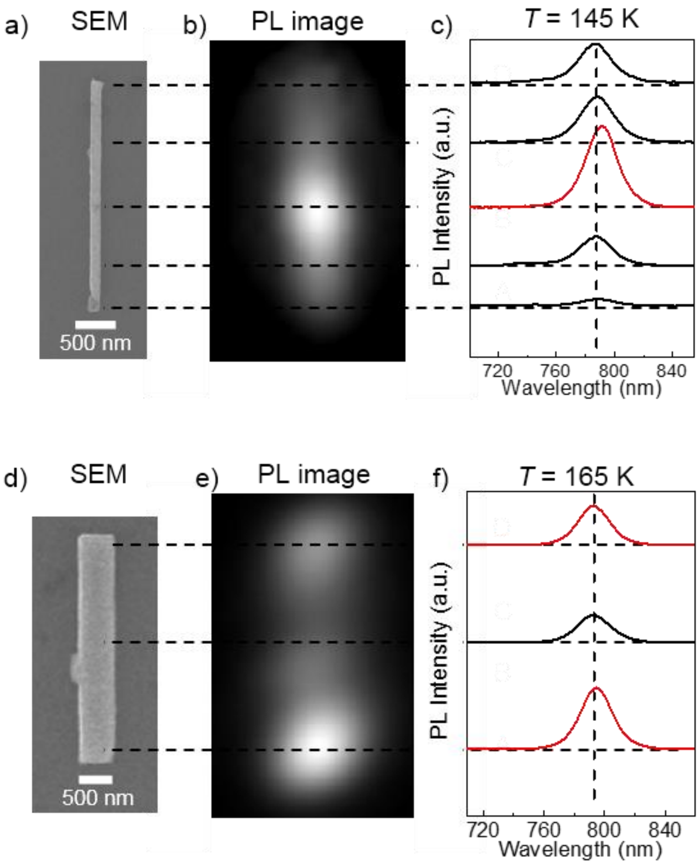

Supplementary Figure 7: Two more examples of the "spotty" luminescence phenomenon in the phase transition temperature region. Fluorescence microscopy of two individual $CH_3NH_3PbI_3$ nanowires in the phase transition temperature region. SEM images of NW#5 (a) and NW#6 (d). Fluorescence images (b,e) and PL spectra (c,f) collected from the different regions of the nanowires at 145 K for NW#5 and 165 K for NW#6 corresponding to the maximum of the PL intensity.

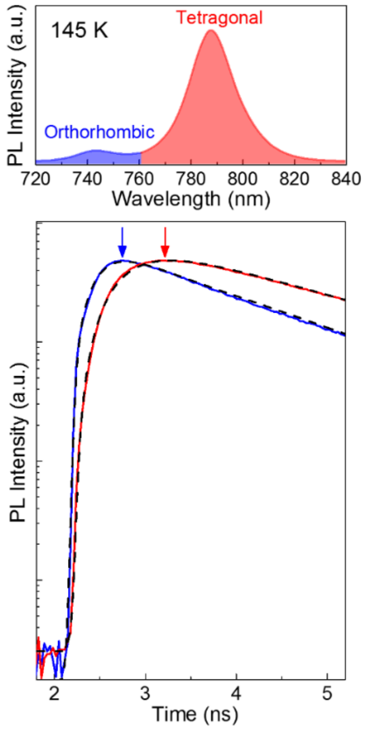

Supplementary Figure 8: PL kinetics of the two phases in the phase transition temperature region. PL kinetics in the orthorhombic phase (blue line) and tetragonal phase (red line) PL bands in the bright region of NW#1 (region C in Figure 4) at 145 K. The filled areas under the curve represent the spectral regions from where the TCSPC data were collected. The dashed lines are multi-exponential fittings. The PL of the tetragonal phase decayed slightly slower than that of the orthorhombic phase at 145 K. However, the PL trace of the tetragonal phase exhibits a pronounced rise component with a time constant of about 0.4 ns. This delayed PL agrees with the charge carrier funnelling from the high-energy orthorhombic phase domains to the lower-energy domains of the tetragonal phase.

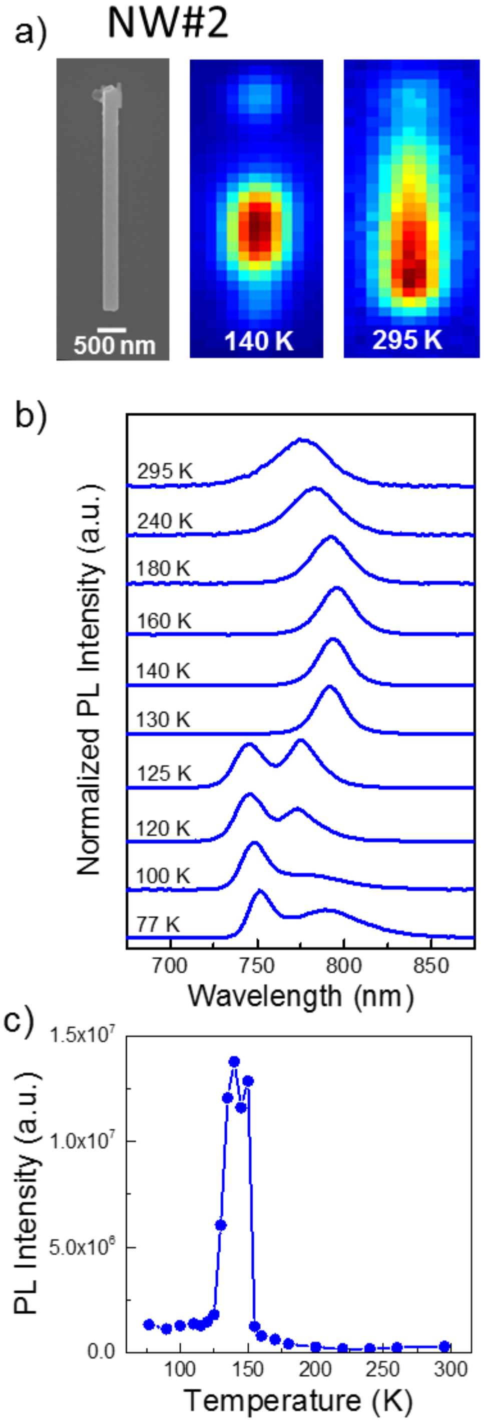

Supplementary Figure 9: SEM and temperature dependent PL characteristics of NW#2. (a) SEM image of NW#2 and its PL images at room temperature and crystal phase transition temperature region (140 K). (b) Normalized PL spectra at different temperatures during cooling. (c) Variation of the integral PL intensity of the nanowire during cooling.

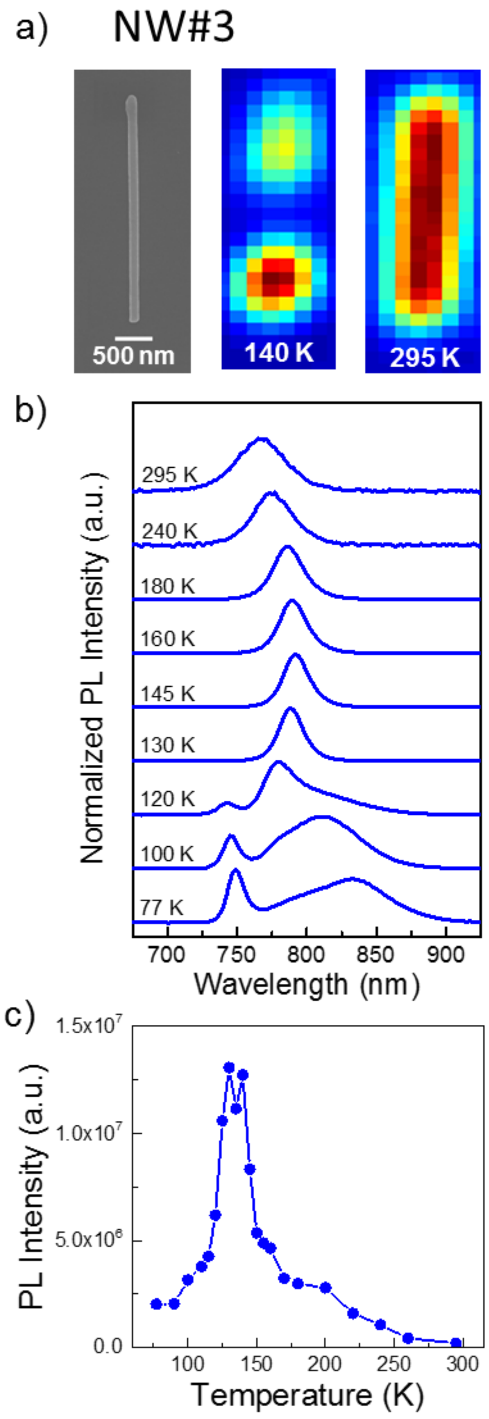

Supplementary Figure 10: SEM and temperature dependent PL characteristics of NW#3. (a) SEM image of NW#3 and its PL image at room temperature and crystal phase transition temperature region (140 K). (b) Normalized PL spectra at different temperatures during cooling. (c) Variation of the integrated PL intensity of the nanowire under cooling.

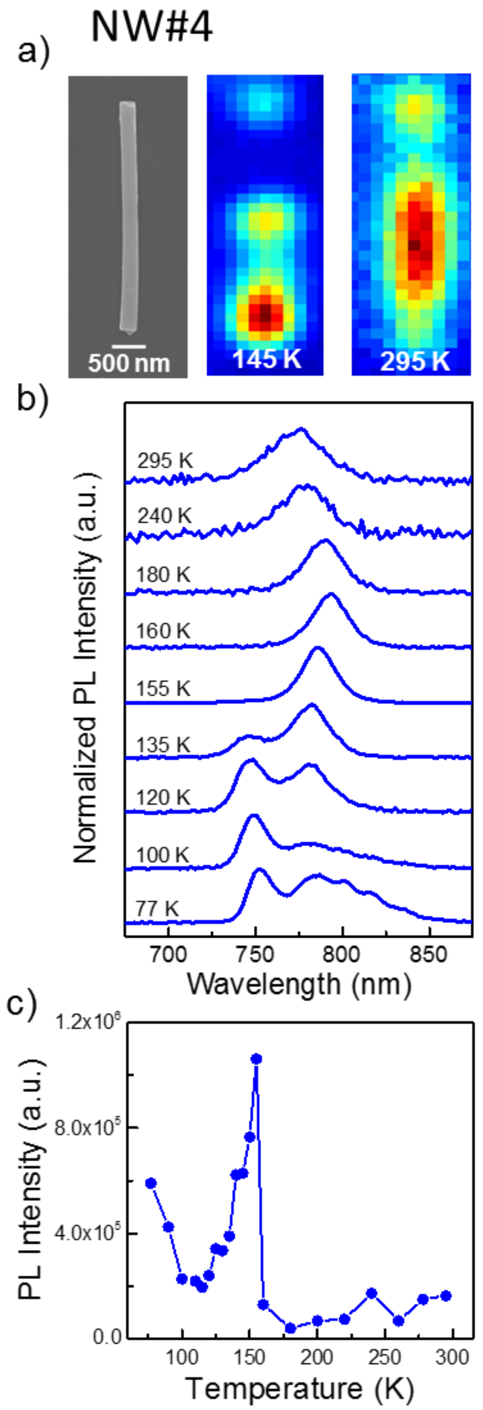

Supplementary Figure 11: **SEM and temperature dependent PL characteristics of NW#4.** (a) SEM and PL image of NW#4 at room temperature and crystal phase transition temperature region (145 K). (b) Normalized PL spectra at different temperatures during cooling. (c) Variation of the integral PL intensity of the NW under cooling.

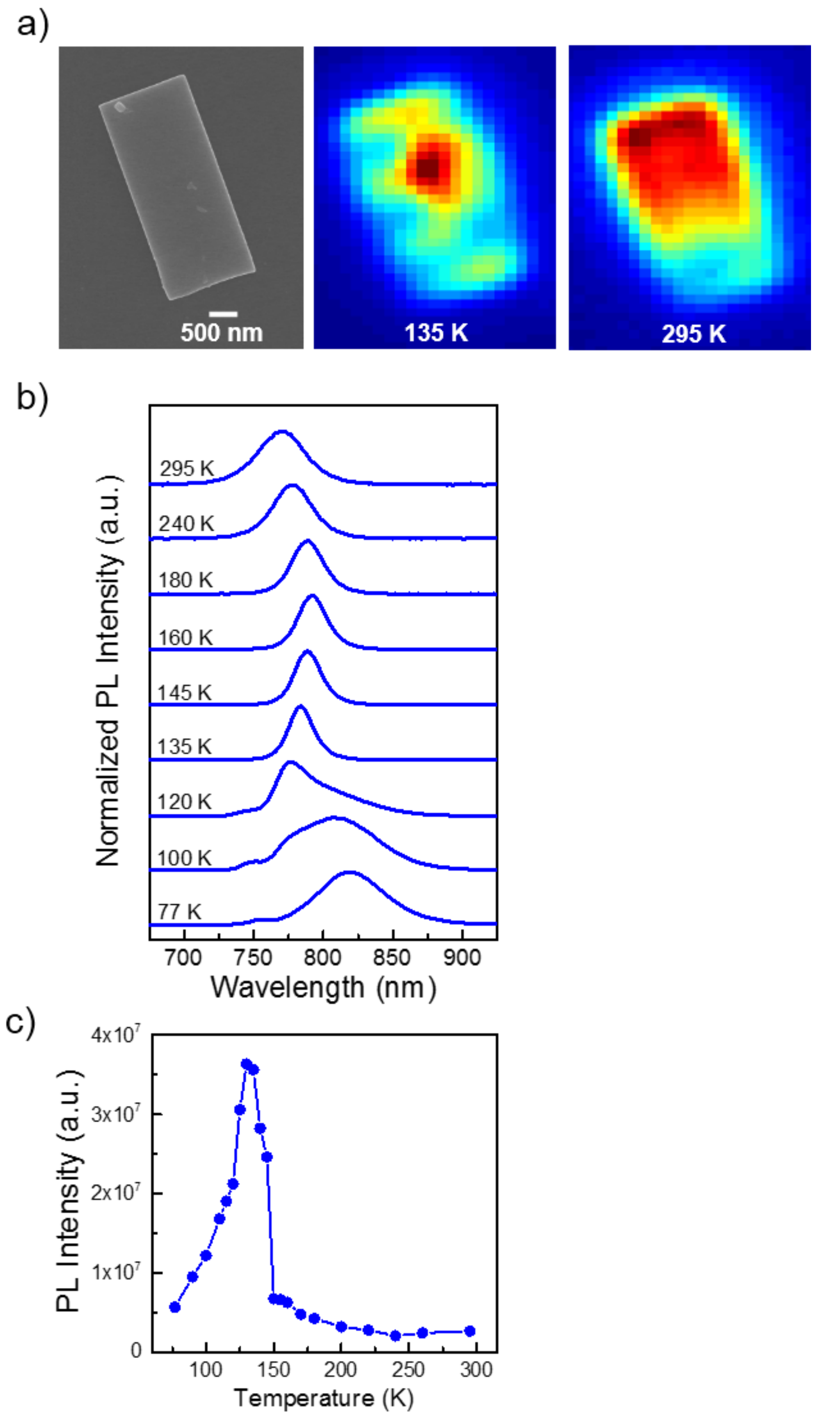

Supplementary Figure 12: SEM and temperature dependent PL characteristics of microplate. (a) SEM of the microplate and its PL images at room temperature and crystal phase transition temperature region (135 K). (b) Normalized PL spectra at different temperatures during cooling. (c) Variation of the integral PL intensity of the microplate under cooling.

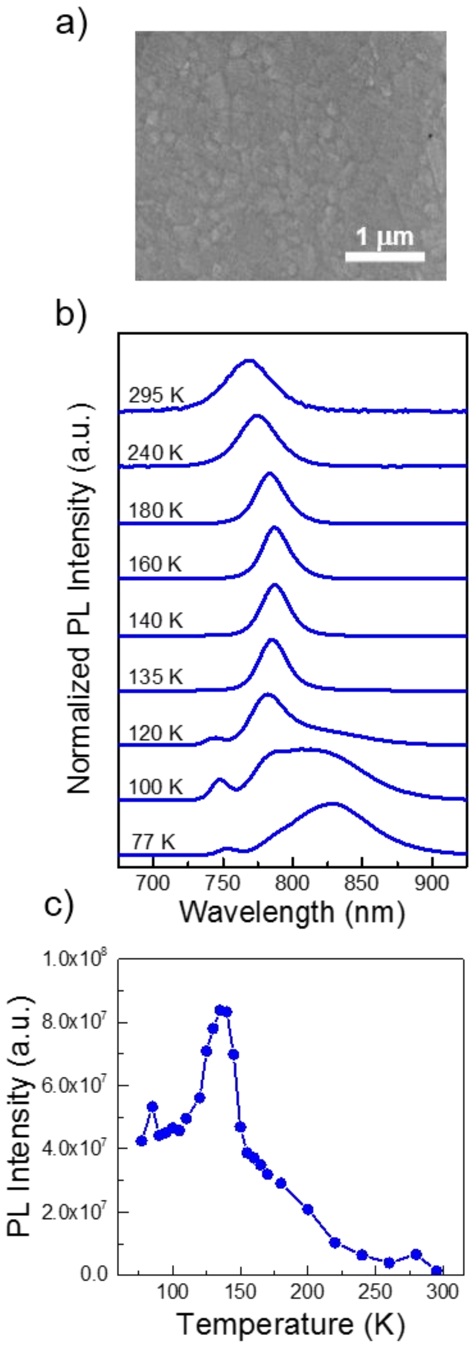

Supplementary Figure 13: SEM and temperature dependent PL characteristics of thin film. (a) SEM image of the film. (b) Normalized PL spectra at different temperatures during cooling. (c) Variation of the integral PL intensity of the film under cooling.

Supplementary References

1. Zhu, H. *et al.* Lead halide perovskite nanowire lasers with low lasing thresholds and high quality factors. *Nat. Mater.* **14,** 636–642 (2015).

2. Baikie, T. *et al.* Synthesis and crystal chemistry of the hybrid perovskite (CH3NH3)PbI3 for solid-state sensitised solar cell applications. *J. Mater. Chem. A* **1,** 5628 (2013).

3. Unger, E. L. *et al.* Chloride in Lead Chloride-Derived Organo-Metal Halides for Perovskite-Absorber Solar Cells. *Chem. Mater.* **26,** 7158–7165 (2014).

4. Tian, Y. *et al.* Mechanistic insights into perovskite photoluminescence enhancement: light curing with oxygen can boost yield thousandfold. *Phys. Chem. Chem. Phys.* **17,** 24978–24987 (2015).

5. Merdasa, A. *et al.* Single Lévy States–Disorder Induced Energy Funnels in Molecular Aggregates. *Nano Lett.* **14,** 6774–6781 (2014).

6. Muls, B. *et al.* Direct Measurement of the End-to-End Distance of Individual Polyfluorene Polymer Chains. *ChemPhysChem* **6,** 2286–2294 (2005).
