# Supplementary material for: Defect-induced local variation of crystal phase transition temperature in metal-halide perovskites
Source: Nat Commun. 2017 Jun 26;8:34. doi: 10.1038/s41467-017-00058-w (PMC5484711; doi:10.1038/s41467-017-00058-w)
Supplement: Supplementary file 3 — Peer Review [file 41467_2017_58_MOESM3_ESM.pdf]

Reviewers' comments:

Reviewer #1 (Remarks to the Author):

The manuscript by Scheblykin et al., reports measurements performed on microcrystals of MAPbI3. Shortly the authors claim that the phase transition between the tetragonal to the orthorhombic phase is not instantaneous but is occurring in domains, which give rise to large spatial inhomogeneity of the sample photoluminescence below the phase transition.
A similar proposal was put forward by Wu et al in Phys.Chem.Chem.Phys.,17,16405-1641 (2015) for crystalline thin films by analysing the PL and the Xray difraction paterns.
Besides the fact that a large portion of the interpretation is not reported for the first time, I find some of the experimental results peculiar. One of them is the flash increase of the PL close to the phase transition. Seeing this I start wondering if a microcrystal with his high surface it is representative of the situation occurring in a real single crystal (Adv. Funct. Mater. 25, 2378–2385 (2015)) where such a behaviors have not been reported, and if we are learning anything from this large variety of samples coming from different laboratories.
For such a study I will require a larger statistics, as I expect that every microcrystal will behave in a sightly different way. Moreover, I will suggest the authors to consider carefully the role of surface defects, recent works show that surface states are present and can be influenced strongly from the surrounding, I will further suggest to check some recent works dealing with laser healing of defects. At last it will be fundamental to provide a better characterization of their samples.

Reviewer #2 (Remarks to the Author):

This manuscript addresses the nature of the low-temperature phase transition between tetragonal and orthorhombic crystal structures exhibited by methylammonium lead iodide (MAPbI3). The transition has been discussed several times previously in the literature but often with slightly differing characteristics depending on the preparation procedure. In my view this variation in results has not been well explained within the perovskite halide field. The authors' contribution to this debate is a careful study the spatial variation in the PL properties of an MAPbI3 nanorod below room temperature. They propose that the spatial variation they observe during the phase transition can be explained by a distribution of defects in the crystal, which influences local strain and determines the transition temperature in different regions. Hence, the two phases can be observed to co-exist over a wider temperature range than may be expected in a perfect crystal, and more generally that the transition range depends on the defect distribution, which is influenced by the crystal preparation procedure. This explanation is well known in material science for first order solid-solid phase transitions and is not particularly remarkable in itself. However, it hasn't been described well as relevant for perovskite halides. The work therefore makes a useful contribution to the growing understanding of phase transitions in perovskite halides and may be of broader interest to materials scientists. My concerns regarding the details of experiment and presentation of the discussion that should be addressed are as follows:

1. The manuscript only shows complete results from a single nanorod and I think this is insufficient. The authors should present further data from repeated experiments in different crystals to support the claim that their proposed mechanism is generally applicable (figure S7 is not sufficient, the full analysis is necessary). Are regions of crystals ever observed that don't exhibit the same trend in defect density distribution and transition temperature?

2. On page three the authors state their motivation for studying an isolated nanostructure is to remove averaging effects and the influence of grain boundaries. I would argue that their measurement technique, using super-resolution imaging, already removes averaging effects

because (presumably) it can probe length scales smaller than crystal grains in some polycrystalline films. Additionally, since thin-films are by far the most important form for perovskites in device applications, the impact of grain boundaries is very important to understand and should not be ignored. I think the work needs to say something about thin-films as well.

3. On page four the authors state that by SEM the thickness of the nanorod is uniform. However SEM is not a very useful technique for judging thickness. AFM data could be presented to confirm whether this is true. Alternatively, presumably it is also possible with the experimental setup to map the optical transmission of the nanorod at a fixed wavelength. How uniform is this map?

4. At the bottom of page five there seems to be an inconsistency with the description of figure 3e. Domains C and B are determined to be >300nm and domain A below <100 nm.

5. There is significant uncertainty in trying to confirm the absence of grain boundaries in the nanorod with such low resolution SEM images. Grain boundaries, or extended defects, might only be observable on the atomic scale so it's quite difficult to prove an object is really a single crystal. I think the manuscript should use more tentative language when discussing the influence of grain boundaries or add more data, using TEM for example, to highlight the crystal quality with more certainty.

6. On page seven the manuscript proposes the mechanism of phase nucleation and growth around defects cannot explain the PL behaviour in the lower temperature region of the phase transition where the orthorhombic phase becomes dominant. I don't fully agree with this. It's likely that as the tetragonal domain size decreases, recombination in the orthorhombic phase competes better with carrier diffusion to a tetragonal domain. This is observed in the increasing intensity of the orthorhombic peak in the same temperature region. No additional explanation is needed.

7. The manuscript contains one or two mentions of defect redistribution with temperature cycling that are not fully explained in the discussion and I don't think are supported by the data. That the region of low PL intensity at room temperature corresponds with the region high defect PL intensity at 77K seems like quite strong evidence that there is no defect re-distribution. These statements need to be clarified.

8. Can the authors add anything to the discussion about which defect species are likely to be more relevant to the phase transition based on previous literature?

Reviewer #3 (Remarks to the Author):

The authors use luminescence spectroscopy and super-resolution imaging to monitor tetragonal to orthorhombic phase transition in stand-alone methylammonium-lead-tri-iodide nanowires and suggest a model to explain drastic photoluminescence enhancement and high spatial inhomogeneity of the photoluminescence by existence of nano-domains with different defect nature and concentrations within a single nanowire object. The authors suggest that tetragonal to orthorhombic phase transition originates in the domains with higher defects and photoluminescence from less-defected tetragonal domains traps the charge carriers generated in the orthorhombic domains, which may explain variations and spreading of the reported phase transition temperatures.

I enjoyed reading the draft provided, although the text would benefit from some minor misprint corrections. In my opinion the experiments we well designed and the claims made by the authors

are mainly valid. I believe after a major revision this work can be considered for publication in Nat. Comm.

The origin of the heterogeneity in phase transition of methylammonium-lead-tri-iodide on a microscale due to variation in strain e.g. structural defects (such as grain boundaries) were previously proposed and the relevant publications are cited in the present work. In my opinion, current study doesn't provide any new evidence of defects influence on the phase transition as no direct characterization of the defects type/ nature were made. The model proposed is very plausible, however without strong experimental confirmation on defect presence, the model provides yet an additional speculation as to the origin of the phase transition in this type of material. That being said, by choosing to look at an individual nano-object, authors not only diminish the uncertainty arising from averaging the nonhomogeneous polycrystalline films, but also provide an easy to work platform to direct characterization of the defect type and nature. Complementing this study with a TEM characterization of the structural defects present in the nanowire in question would provide adequate evidence to the statements made in this work. Given that such evidence is provided I would be happy to further review this work.

Point by point reply to reviewers.

We are very grateful to the reviewers for their valuable comments on our manuscript which helped up to make our case stronger. Please, see below our answers and description of the changes in the manuscript. All important changes in the manuscript text are highlighted by blue font color.

Reviewers' comments:

Reviewer #1 (Remarks to the Author):

Comment 1-1. The manuscript by Scheblykin et al., reports measurements performed on microcrystals of $MAPbI_3$. Shortly the authors claim that the phase transition between the tetragonal to the orthorhombic phase is not instantaneous but is occurring in domains, which give rise to large spatial inhomogeneity of the sample photoluminescence below the phase transition. A similar proposal was put forward by Wu et al in Phys.Chem.Chem.Phys.,17,16405-1641 (2015) for crystalline thin films by analyzing the PL and the X-ray diffraction patterns. Besides the fact that a large portion of the interpretation is not reported for the first time, I find some of the experimental results peculiar. One of them is the flash increase of the PL close to the phase transition. Seeing this I start wondering if a microcrystal with his high surface it is representative of the situation occurring in a real single crystal (Adv. Funct. Mater. 25, 2378–2385 (2015)) where such a behaviors have not been reported, and if we are learning anything from this large variety of samples coming from different laboratories.

Reply 1-1

We thank the reviewer for this comment. Here we feel that we probably did not formulate the novelty of our study clear enough, so we try to do it better in the revised version. We are indeed familiar with these two papers which were referred to in the original submission. As we mentioned in the introduction, the proposal of having domains or high level of disorder in the phase transition region is not new.

Our contribution to this is that we actually observed the nano-domains coexisting experimentally at the level of single sub-micrometer MAPbI3 wires. As for the PL enhancement, it has also been reported previously in thin films as we acknowledged. Just the fact of PL enhancement is not new, however, its presence in such small objects as highly-crystalline individual nanowire forced us to think about its nature and come up with the idea that the local phase transition temperature is simply determined by the chemical nature and concentration of the defects in the particular local (scale of tens – hundreds of nanometers) region. We are quite convinced that this idea has not been around in the perovskite community although theory of this is known for years.

There are actually not so many data in the literature where PL yield was measured as a function of temperature with small enough temperature steps. We do not think that the possible absence of significant PL enhancement in some cases contradicts to our model. The following sentence was added to the text on page 9: "*Note, that small extent or even absence of PL enhancement, which some reports seem to show,*[53] *by no means undermines the generality of our model. It only*

implies that the defects which were determining the phase transition dynamics in those samples were not the ones which were crucial for their PL quantum yield. In this case the local PL yield becomes insensitive to local crystal phase."

In the revised version of the manuscript we present data for several more nanowires, a plate and a film and saw that the extent of the PL enhancement effect can vary quite a bit. So, what we are learning from different samples (also from the literature data) is that phase transition can happen over a wide range of temperatures which makes perfect sense in the framework of our model where not only the strain, but also defects influence the phase transition locally.

As we understand that one of the objective of the work on macroscopic single crystals mentioned by the reviewer (Ref. 10 in the revised manuscript) was to demonstrate that the PL intensity is increasing by two orders of magnitude by cooling from room temperature to 5K (Figure 2c in Ref. 10). Due to this specific focus the temperature dependence of PL was naturally presented there in a semi-logarithmic scale *vs* inversed temperature. Our study focuses specifically on the narrow temperature region of the phase transition around 160 K. Unfortunately, we find it difficult to directly compare our results to that of Figure 2c in Ref. 10 since the sharp enhancement could occur within a very narrow interval on 1/T scale: between x=1/T= 0.0063 (1/160 K) and x =1/T = 0.0071 (140 K) in Figure 2c (see a zoom to this figure below).

We agree that the effect of PL enhancement seems not visible in Fig. 2 at first glance, but we argue that this might be due to the specific data representation and a very broad temperature range of interest. However, we want to emphasize that the data do not contradict each other in any way even if there was no PL enhancement indeed (see page 9 in the revised version).

With that said, there still seems to be indications of PL enhancement at the transition temperature region at close inspection of Fig. 2c, which we have tried to demonstrate in the figure below with notes.

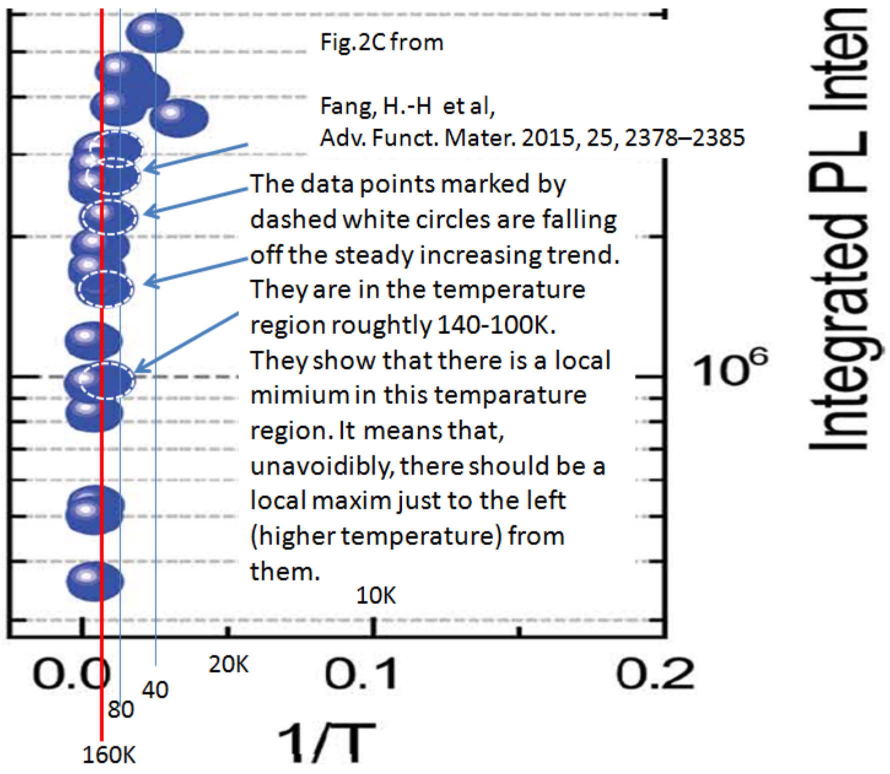

Comment 1-2. For such a study I will require a larger statistics, as I expect that every microcrystal will behave in a slightly different way. Moreover, I will suggest the authors to consider carefully the role of surface defects, recent works show that surface states are present and can be influenced strongly from the surrounding, I will further suggest to check some recent works dealing with laser healing of defects. At last it will be fundamental to provide a better characterization of their samples.

Reply 1-2. We totally agree with the reviewer that a larger statistics is needed. Experiments on individual objects are not easy, however, we are happy to present new experimental data obtained for 3 new nanowires, 1 microplate and a film in our revised version. Figure 1 was changed in order to incorporate the new data where the full set of experimental data on these new objects can be found in SI). All this data is in agreement with that presented in the original submission. Namely, in all cases we observed PL enhancement (even for the film and the micro-plate), although the extent of enhancement varied. As the reviewer expected, the PL enhancement in the film was less pronounced than in individual wires just because of the averaging effect since in each grain of the film the phase transition occurs at different temperature.

The particular procedure we employed for fabrication of our samples is known from the literature to produce high quality wires and plates.[Ref. 8] $MAPbI_3$ wires and plates have been characterized in that paper by X-ray diffraction, SEM and TEM. SEM images were added to the revised version of the manuscript and SI shows that the wires and the plate have clear crystallographic faces that is indicative of their high crystal quality. STEM image (added to the

revised version, see Figure S1) also shows that nanowires are quite homogeneous through their thickness.
Our data does not allow us to distinguish between the surface and bulk defects. Both of them can influence the phase transition temperature and the model we proposed (based on known theory) does not differ between surface or bulk defects. Although more experimental studies are needed here, for the low-temperature measurements we should keep samples in an evacuated chamber that restricts our ability to change their environment. We mention possible effect of the surface states on the transition temperature in the revised version now (page 9) and added several references on works dealing with laser healing of defects (see Ref. 19-22 in the revised version) according to the reviewer recommendations.

Reviewer #2 (Remarks to the Author)**:**

This manuscript addresses the nature of the low-temperature phase transition between tetragonal and orthorhombic crystal structures exhibited by methylammonium lead iodide (MAPbI3). The transition has been discussed several times previously in the literature but often with slightly differing characteristics depending on the preparation procedure. In my view this variation in results has not been well explained within the perovskite halide field. The authors' contribution to this debate is a careful study the spatial variation in the PL properties of an MAPbI3 nanorod below room temperature. They propose that the spatial variation they observe during the phase transition can be explained by a distribution of defects in the crystal, which influences local strain and determines the transition temperature in different regions. Hence, the two phases can be observed to co-exist over a wider temperature range than may be expected in a perfect crystal, and more generally that the transition range depends on the defect distribution, which is influenced by the crystal preparation procedure. This explanation is well known in material science for first order solid-solid phase transitions and is not particularly remarkable in itself. However, it hasn't been described well as relevant for perovskite halides. The work therefore makes a useful contribution to the growing understanding of phase transitions in perovskite halides and may be of broader interest to materials scientists. My concerns regarding the details of experiment and presentation of the discussion that should be addressed are as follows:

Comment 2-1. The manuscript only shows complete results from a single nanorod and I think this is insufficient. The authors should present further data from repeated experiments in different crystals to support the claim that their proposed mechanism is generally applicable (figure S7 is not sufficient, the full analysis is necessary). Are regions of crystals ever observed that don't exhibit the same trend in defect density distribution and transition temperature?

Reply 2-1. We absolutely agree with the reviewer (see also Reply 1-2). Data on four more different individual objects and a film has been added to the manuscript. Please, see modified Fig. 1 in the main text, and Section 9 which was added to SI. So far all objects we studied possessed PL enhancement in the phase transition temperature region and "spotty" luminescence pattern to some extent.

Comment 2-2. On page three the authors state their motivation for studying an isolated

nanostructure is to remove averaging effects and the influence of grain boundaries. I would argue that their measurement technique, using super-resolution imaging, already removes averaging effects because (presumably) it can probe length scales smaller than crystal grains in some polycrystalline films. Additionally, since thin-films are by far the most important form for perovskites in device applications, the impact of grain boundaries is very important to understand and should not be ignored. I think the work needs to say something about thin-films as well.

Reply 2-2. We agree with the reviewer and have added measurements on a thin film. Preparation procedure was added to the method section. The results are similar, however, the relative PL enhancement is not so pronounced and the region of enhanced PL is stretched over larger temperature range as one would expect for an inhomogeneous system where properties of different micro and nanocrystals are averaged out. We have added a discussion where individual crystals and the films are compared, see page 9.

Comment 2-3. On page four the authors state that by SEM the thickness of the nanorod is uniform. However SEM is not a very useful technique for judging thickness. AFM data could be presented to confirm whether this is true. Alternatively, presumably it is also possible with the experimental setup to map the optical transmission of the nanorod at a fixed wavelength. How uniform is this map?

Reply 2-3. We agree that SEM does not provide much information about the thickness of the wires. However, we are quite convinced that the wires posses quite uniform thickness as well as the width (the latter we see in SEM). The particular procedure we employed is known from the literature to produce high quality wires and plates.[Ref. 8] $MAPbI_3$ wires and plates have been characterized in that paper by X-ray diffraction, SEM and TEM. After observing many perovskite crystals by SEM, our experience tells us that we reproduce the samples reported in the mentioned publication very well. As for the absorption measurements, unfortunately, their thickness is far too small to be able to judge the uniformity of absorption using optical microscopy where lateral resolution is not better than 500 nm. Using AFM is of course a good idea, although technically it is difficult for us to do there measurements on individual objects in combination with PL microscopy.

In revised version of SI we show now an image of one of the wires which was tilted. The image shows that the cross section is rectangular (Figure S1(b)). We also tried to use STEM (scanning transmission electron microscopy) and present some data in SI (Figure 1), see also our Reply 3-2.

Comment 2-4. At the bottom of page five there seems to be an inconsistency with the description of figure 3e. Domains C and B are determined to be >300nm and domain A below <100 nm.

Reply 2-4. We are grateful to the reviewer for careful reading. The error was corrected.

Comment 2-5. There is significant uncertainty in trying to confirm the absence of grain boundaries in the nanorod with such low resolution SEM images. Grain boundaries, or extended

defects, might only be observable on the atomic scale so it's quite difficult to prove an object is really a single crystal. I think the manuscript should use more tentative language when discussing the influence of grain boundaries or add more data, using TEM for example, to highlight the crystal quality with more certainty.

Reply 2-5. We agree with the reviewer, it is difficult to prove that the objects do not have grain boundaries at any chosen scale. Moreover, at the phase transition region there must be some kind of boundaries between different crystal phases. What we can tell is that our objects are more homogeneous than films and have well defined crystallographic faces in comparison with randomly shaped individual grains in films. We made this more clear in the revised version.

We also stressed in the revised version that the method used for growing the crystals is well-established [Ref. 8] and SEM images of our wires looks the same as the ones reported in the original paper.[Ref. 8] We feel to carry out the full analysis again to prove the high crystallinity is not necessary.

In addition, high-resolution TEM images are unfortunately very difficult to obtain because of the electron beam induced degradation of the material (see Ref. 8). In any case, in our model we talk about defects as the reason for spreading the phase transition over a temperature range. Grain boundaries are generally speaking also defects contributing to the phase transition dynamics. So, we realized now that it is not necessary for us to stress that there are no boundaries. The results on the film (both our own and data from literature [Ref. 33 in the revised version]) clearly demonstrate that materials with more grain boundaries (defects) possess prolonged phase transition in comparison with more uniform samples (like nanowires).

In the revised version we use a more careful language regarding this issue and explicitly discuss crystallinity and quality of wires vs films on page 3.

Comment 2-6. On page seven the manuscript proposes the mechanism of phase nucleation and growth around defects cannot explain the PL behaviour in the lower temperature region of the phase transition where the orthorhombic phase becomes dominant. I don't fully agree with this. It's likely that as the tetragonal domain size decreases, recombination in the orthorhombic phase competes better with carrier diffusion to a tetragonal domain. This is observed in the increasing intensity of the orthorhombic peak in the same temperature region. No additional explanation is needed.

Reply 2-6. We thank the reviewer for this comment. We realized that we forgot to mention in the original text that initially we assumed a very efficient diffusion of charges over the whole wire. With this assumption, there is no real "competition" between the two phases: as soon as the tetragonal phase exists, it should absorb all charges. In order to get the "competition", exactly as suggested by the reviewer, we need to assume that the charge migration is not so fast and efficient. We have modified the text to make our logic clear (see page 8) Therefore, our explanation is exactly the one proposed by the reviewer. We hope the text is clear on this now.

Comment 2-7. The manuscript contains one or two mentions of defect redistribution with temperature cycling that are not fully explained in the discussion and I don't think are supported by the data. That the region of low PL intensity at room temperature corresponds with the region

high defect PL intensity at 77K seems like quite strong evidence that there is no defect re-distribution. These statements need to be clarified.

Reply 2-7. We thank the reviewer for this comment. We believe that the reviewer noticed "defect redistribution" in the very last paragraph of the conclusion section. Admittedly, the language used there was not appropriate. We meant that the defects become located in a particular phase (re-distribution between phases), but we did not want to say that defects somehow move in space during the cooling-heating cycles. We have modified the sentence to avoid misunderstanding (see page 10).

Comment 2-8. Can the authors add anything to the discussion about which defect species are likely to be more relevant to the phase transition based on previous literature?

Reply 2-8. This is a very important but a very difficult task. There are many defects sites (vacancies, substitutions and interstitials). The only criteria we can use to select possible defects is that there defects must quench PL. If we assume a Shockley-Read-Hall non-radiative processes, then the defects should have relatively deep levels in the bandgap. According the paper Yin, W.-J., Shi, T. & Yan, Y. Unusual defect physics in $CH_3NH_3PbI_3$ perovskite solar cell absorber. *Appl. Phys. Lett.* **104,** 063903 (2014) (Ref. 26) such deep traps are I_{MA}, I_{Pb}, Pb_i, Pb_I. We have mentioned this in the revised version of the paper.

Reviewer #3 (Remarks to the Author):

The authors use luminescence spectroscopy and super-resolution imaging to monitor tetragonal to orthorhombic phase transition in stand-alone methylammonium-lead-tri-iodide nanowires and suggest a model to explain drastic photoluminescence enhancement and high spatial inhomogeneity of the photoluminescence by existence of nano-domains with different defect nature and concentrations within a single nanowire object. The authors suggest that tetragonal to orthorhombic phase transition originates in the domains with higher defects and photoluminescence from less-defected tetragonal domains traps the charge carriers generated in the orthorhombic domains, which may explain variations and spreading of the reported phase transition temperatures.

I enjoyed reading the draft provided, although the text would benefit from some minor misprint corrections. In my opinion the experiments we well designed and the claims made by the authors are mainly valid. I believe after a major revision this work can be considered for publication in Nat. Comm.

Comment 3-1. The origin of the heterogeneity in phase transition of methylammonium-lead-tri-iodide on a microscale due to variation in strain e.g. structural defects (such as grain boundaries) were previously proposed and the relevant publications are cited in the present work. In my opinion, current study doesn't provide any new evidence of defects influence on the phase transition as no direct characterization of the defects type/ nature were made.

Response 3-1.

Although indeed the influence of strain induced by grain boundaries has been proposed to influence the phase transition by stabilizing one phase or another, the role of defects in general (a grain boundary is also a defect) has not been discussed. Therefore, we consider our contribution here in being able to connect the existing experiments at the bulk level and our novel experiment at the level of individual nano-micro crystals with the existing theory of phase transitions in solids.

The PL enhancement effect was observed before but never explained. This effect is crucial here. We realized, and this is our contribution, that it provides direct experimental connections between the defects (defects quench PL) and the phase transition. As far as we know, this is the first explanation of the PL enhancement in the phase transition region provided. We are the first who connected location of PL quenching defects and location of the phase nucleation sites. We also showed that PL quantum yield and spectrum are the properties which are able (due to the connection to the defects) to tell us a lot about the phases in the material. In the revised version we put some efforts to make the novelty of our work more clear.

Comment 3-2.

The model proposed is very plausible, however without strong experimental confirmation on defect presence, the model provides yet an additional speculation as to the origin of the phase transition in this type of material.

That being said, by choosing to look at an individual nano-object, authors not only diminish the uncertainty arising from averaging the nonhomogeneous polycrystalline films, but also provide an easy to work platform to direct characterization of the defect type and nature.

Complementing this study with a TEM characterization of the structural defects present in the nanowire in question would provide adequate evidence to the statements made in this work. Given that such evidence is provided I would be happy to further review this work.

Reply 3-2.

We agree with the reviewer that identifying the exact chemical nature by our indirect method is impossible; we have added some discussion on this to the revised text (see Reply 2-8). However, the presence of defects as such in $MAPbI_3$ is well documented: we know there are defects, but which ones in the particular sample, we do not really know.

As for the TEM characterization, we agree that it would be awesome to be able to see each atom of the nanowire, see the defects and may be even see formation of a particular phase around a particular defect. However, for us to do such a “dream study” is very difficult if not impossible. The main reason is that for getting higher resolution for thick (500 nm) nanowires one needs to use highly energetic electron beams which was shown to degrade $MAPbI_3$.[Ref. 8]. Even low irradiation dozes were shown to introduce defects. Another problem is that technically we are not able to study the same nanowire by TEM which we observed by PL microscopy (because of the sample substrates, microscope cover slip vs TEM grid). So, unfortunately, correlation between

PL and TEM is not possible today. We still thank the reviewer for this comment and hope to design experiments towards using TEM in phase transition studies in the future; for that we probably need to look at very small nano objects (<100 nm in thickness) which are more easy to look through.

However, in response to the reviewer's concern and also responding to Reviewer 1, we have added several more objects to our study demonstrating nice clear crystallographic faces on SEM images showing absence of major structural inhomogeneities in comparison with clearly inhomogeneous films. Although we cannot exclude local inhomogenieties at the atomic level, they are most probably present, it will not change our model and conclusions. The main difference is that films consist of randomly shaped crystallites of 100 – 300 nm in size while nano-wires are uniformly looking rectangular objects 4000 nm long and hundreds of nanometers thick. The regular rectangular shape of the wire means that the directions of crystallographic axes are well defined and constant for the whole object. This immediately tells us that in terms of number and size of structural defects, wires and plates must be much better than even individual grains in film.

We also want to point out that the synthesis method we used is well documented and known to deliver well-defined perovskites rods and plates as has been characterized by TEM and X-ray diffraction.[Ref. 8] We stressed this more in the revised introduction (page 3). See also Response 2-5 on a somewhat related question.

To summarize, while still not using TEM due to the technical reasons, we used STEM (scanning transmission electron microscopy). The image presented in SI (Figure S1) shows that we are not able to see though thick crystals, while thin crystals appear quite homogeneous through their thickness.

Reviewers' comments:

Reviewer #1 (Remarks to the Author):

The authors have provided a convincing answer to the reviewer concerns and have added some experimental data to the original set. Overall, the current version of the manuscript, has the quality to be publish in Nature Communications.

Reviewer #2 (Remarks to the Author):

I think the authors have adequately addressed the concerns raised during the review. The conclusions are now clearer and will be of interest to the field.

Reviewer #3 (Remarks to the Author):

The explanations provided by the authors and the changes made in the MS and SI are sufficient. I think the work is now ready for publication and will constitute an excellent new contribution to the understanding of perovskite materials.
